# The effects of household wealth on HIV prevalence in Manicaland, Zimbabwe – a prospective household census and population-based open cohort study

**DOI:** 10.7448/IAS.18.1.20063

**Published:** 2015-11-20

**Authors:** Nadine Schur, Adrian Mylne, Phyllis Mushati, Albert Takaruza, Helen Ward, Constance Nyamukapa, Simon Gregson

**Affiliations:** 1 Department of Infectious Disease Epidemiology, Imperial College London, London, UK; 2 Biomedical Research and Training Institute, Harare, Zimbabwe

**Keywords:** HIV prevalence, household assets, Manicaland, occupation, poverty, socio-economic status, wealth, Zimbabwe

## Abstract

**Introduction:**

Intensified poverty arising from economic decline and crisis may have contributed to reductions in HIV prevalence in Zimbabwe.

**Objectives:**

To assess the impact of the economic decline on household wealth and prevalent HIV infection using data from a population-based open cohort.

**Methods:**

Household wealth was estimated using data from a prospective household census in Manicaland Province (1998 to 2011). Temporal trends in summed asset ownership indices for sellable, non-sellable and all assets combined were compared for households in four socio-economic strata (small towns, agricultural estates, roadside settlements and subsistence farming areas). Multivariate logistic random-effects models were used to measure differences in individual-level associations between prevalent HIV infection and place of residence, absolute wealth group and occupation.

**Results:**

Household mean asset scores remained similar at around 0.37 (on a scale of 0 to 1) up to 2007 but decreased to below 0.35 thereafter. Sellable assets fell substantially from 2004 while non-sellable assets continued increasing until 2008. Small-town households had the highest wealth scores but the gap to other locations decreased over time, especially for sellable assets. Concurrently, adult HIV prevalence fell from 22.3 to 14.3%. HIV prevalence was highest in better-off locations (small towns) but differed little by household wealth or occupation. Initially, HIV prevalence was elevated in women from poorer households and lower in men in professional occupations. However, most recently (2009 to 2011), men and women in the poorest households had lower HIV prevalence and men in professional occupations had similar prevalence to unemployed men.

**Conclusions:**

The economic crisis drove more households into extreme poverty. However, HIV prevalence fell in all socio-economic locations and sub-groups, and there was limited evidence that increased poverty contributed to HIV prevalence decline.

## Introduction

There is a long-held belief that poverty has driven HIV epidemics around the world [1]. This is reflected in reports by the World Bank [2], the United Nations Joint Programme on HIV/AIDS (UNAIDS) [3] and scientific publications [4–7]. It is believed that poverty results in adoption of high-risk sexual behaviours and hence increases the risk of acquiring HIV. Specific risky sexual practices that have been associated with poverty include earlier sexual debut [8,9] and reliance on transactional sex or sex work in order to generate income [7,10]. Other studies have challenged the idea that poverty fuels HIV epidemics and have shown that HIV prevalence can be higher in wealthier populations [11–15]. Richer individuals may engage in other risky sexual practices, such as more regular or casual sex partners [1,14], and those who are infected might survive longer due to greater access to treatment and care and better diet [16]. Gender often plays an important role in the poverty/wealth-HIV relationship. While poverty and lack of property rights can draw women into transactional sex, wealthier men with greater social autonomy can afford to pay for multiple sexual partners [17]. Finally, some studies have suggested that relative wealth can be associated with higher HIV risk initially but may become a protective factor as epidemics mature [1,18–23]. However, it is unclear whether this trend will occur in circumstances of rapid macro-economic change.

Zimbabwe has experienced one of the world's largest HIV epidemics but, since 1997, HIV prevalence in the population aged 15 years and above has fallen steadily from over 25% in 1997 to less than 15% in 2011 [24,25]. The country has also undergone dramatic economic changes during this period. Gross domestic product (GDP) *per capita* began to fall around 2003, followed by an escalating economic decline that culminated in record levels of hyper-inflation and the collapse of the local currency early in 2009. Subsequently, there has been a modest recovery in the economy [26,27]. There is controversy as to whether the economic decline contributed to the fall in HIV prevalence in the country [28–33].

The financial crisis in Zimbabwe offers a unique opportunity to study the impact of economic change on HIV infection rates. In this paper, we investigate how the country's economic decline affected household wealth and patterns of association between prevalent HIV infection and socio-economic location of residence, household wealth and occupation in Manicaland, eastern Zimbabwe, using data collected from 1998 to 2011 in a large longitudinal household survey.

## Methods

### Study area and Manicaland study

As of the 2012 national census [34], almost 13 million people lived in Zimbabwe, out of which 1.5 million were people living with HIV [with around 38% receiving antiretroviral therapy (ART)], 69,000 became newly infected and 46,000 died due to AIDS [35]. During the economic crisis, the health system almost collapsed, with high staff turnover and serious drug shortages. Antiretroviral treatment and prevention of mother-to-child transmission services were introduced first in the mid-2000s, but because of the national economic crisis only became widely available towards the end of the decade. Other key health problems in the country are malaria and tuberculosis. Non-communicable diseases such as cancer, heart failure and diabetes are now on the increase as the population ages.

**Figure 1:**
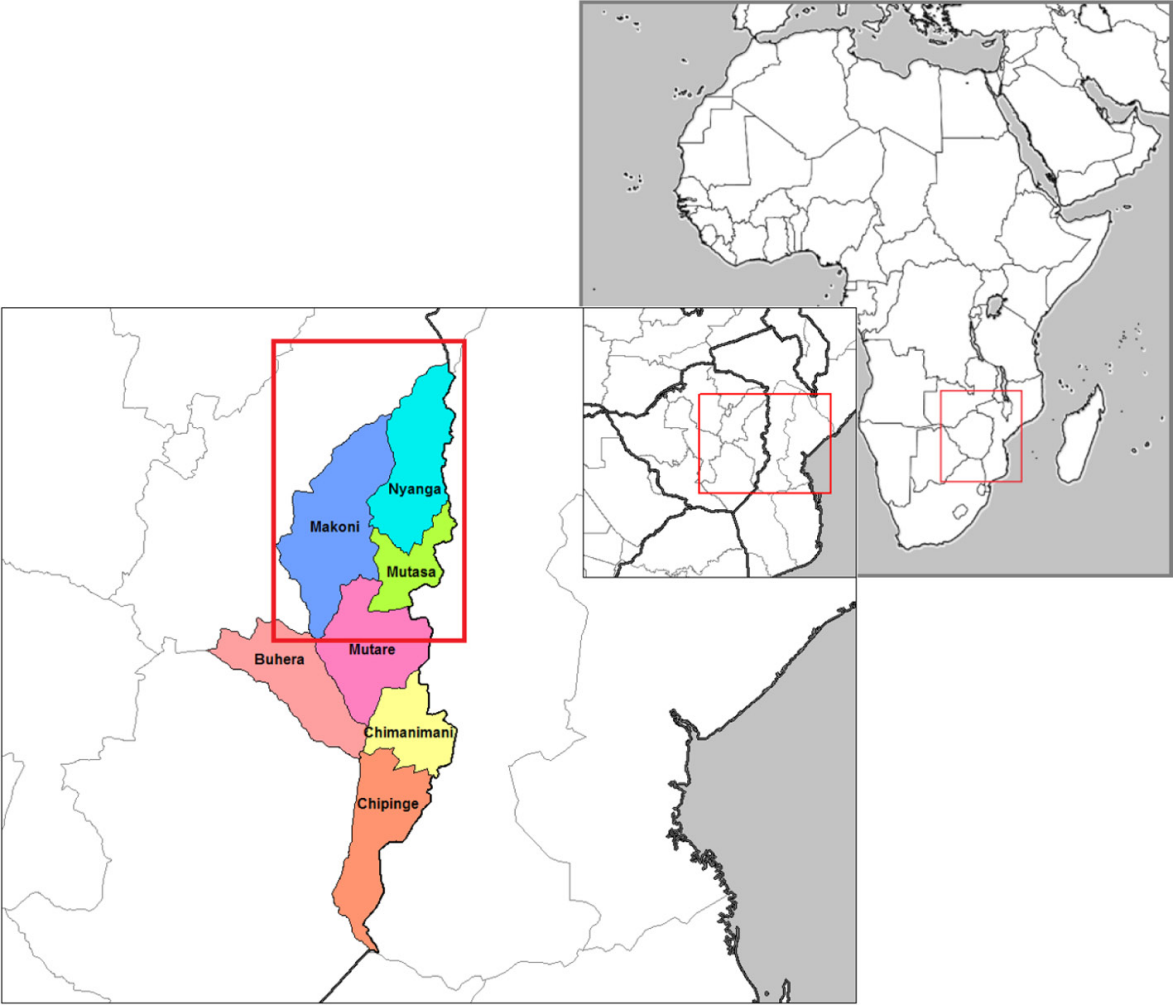
Study districts.

The Manicaland HIV/STD Prevention Project (Manicaland Study) is a collaborative scientific research programme in predominantly rural areas of eastern Zimbabwe (see Figure 1) that investigates trends in the spread of the HIV epidemic and its impact. Full details of the study can be found elsewhere [32,36]. In short, a large prospective population-based open cohort study was initiated in July 1998, covering 12 communities from four different socio-economic strata – small towns (population < 10,000), agricultural estates (tea and forestry plantations), roadside business centres (RBCs) (small settlements close to major tarred roads with markets and few shops) and subsistence farming areas (SFAs) (rural villages) in Manicaland Province. Five rounds were completed: 1998 to 2000 (Round 1), 2001 to 2003 (Round 2), 2003 to 2005 (Round 3), 2006 to 2008 (Round 4) and 2009 to 2011 (Round 5). Questionnaires from all rounds are available online via the project website [36].

### Household census

In each round, all pre-existing and new households within the study area were registered in an initial household census and additional data were collected on household characteristics such as demographic factors and household structure. Household asset information was also obtained for all new households at each round, while data on assets of follow-up households were collected during Rounds 3, 4 and 5. No information on household assets was collected in Round 2 mainly due to financial constraints. The analysis of household wealth presented here focuses on rounds with asset information on new as well as follow-up households (i.e. omitting Round 2).

### Adult individual survey

Individual eligibility criteria varied over survey rounds and are described in more detail elsewhere [32,33,36]. Adults registered during the household censuses and eligible for an interview were requested to answer questions on demographic and behavioural-related factors after written informed consent was obtained. In addition, dried blood spots were collected for anonymous HIV serotesting during each round. Testing was performed using highly sensitive and specific antibody dipstick assays [32]. To create more comparable datasets over survey rounds, only adults aged 15 to 44 years were used in the individual-level analyses presented here.

Prior ethical approval was granted by Medical Research Council of Zimbabwe, Biomedical Research and Training Institute's Institutional Review Board and the Imperial College Research Ethics Committee.

### Socio-economic status

Individual socio-economic status was estimated from data on asset ownership in households of residence. Summed asset ownership scores were created from the available variables in the household questionnaire, for all assets combined and, separately, for sellable and non-sellable assets [31,37]. Sellable assets included radios, televisions, bicycles, motorbikes and cars, while water and electricity supply, toilet facilities, housing structure and floor type were considered as non-sellable assets.

For calculation of the summed asset ownership scores, ordinal asset variables were transformed into values between 0 and 1. For example, a score of 0 was assigned for a natural type floor (e.g. sand), 0.5 for a rudimentary type (e.g. planks) and 1 for finished floors (e.g. cement). The values of all (transformed) variables were summed and divided by the total number of assets to create the summed asset ownership scores. The absolute wealth of a household was based on equally spaced cutoffs of the overall asset score distribution at 0, 0.2, 0.4, 0.6 and 0.8. Household wealth scores were merged with individual survey data to determine associations with HIV infection risk.

### Statistical analysis

Household-level analyses were conducted to assess trends in wealth over time. Panel data regression models with fixed (or all time-invariant) random effects, accounting for household-specific unobserved heterogeneity (the time-constant within-household variation), and robust variance estimators were fitted to take into account that part of the data stem from the same households observed at multiple rounds and, hence, are not independent. Of note, at least two-thirds of households in each round were also interviewed in the following round and, even though the dataset was not completely balanced (meaning that all households would have been present in all rounds), it was still possible to run panel data regression models. For comparison reasons, an additional analysis on the subset of the balanced data was carried out as well.

Quadratic B-spline functions of the survey year with knots set at 1998, mid-2005 and 2011 were employed to assess for non-linear time trends. Preliminary analyses showed that these spline functions fitted the data as well as (or better than) cubic spline functions, functions with more knots or differently placed knots. Analyses were conducted for combined sellable and non-sellable summed asset ownership scores and separately for each study site type (socio-economic location).

Associations between wealth and HIV serostatus by survey round were measured for men and women separately using multivariate logistic regression analyses. The analyses took into account possible clustering at the household level, whereby multiple people of different risk profiling may live within the same household and therefore have the same wealth. Age, educational level, marital status and study site type were included as confounding factors. In addition, models were adjusted for individual occupation with current unemployment as baseline and professional labour, unskilled labour in the informal sector and unskilled labour in other sectors as categories. The results of the above analyses were compared over the different rounds to assess for differences and dynamics over time.

All analyses were conducted in Stata/SE 12.1 for Windows.

## Results

### Household-level analysis

The total number of households enumerated in the census increased from 8374 during the initial round (1998 to 2000) to 14,728 in 2009 to 2011. The participation rate was consistently high (over 90%) but decreased somewhat over time. Incomplete asset information varied between 0.2 and 2.0% and households with incomplete information were excluded from analyses. This process resulted in total numbers of households with complete asset data of 8201 at Round 1; 9360 at Round 3; 12,309 at Round 4; and 13,335 at Round 5.

No consistent trends for all assets over time were observed. While possession of certain assets varied only slightly (e.g. households with electricity), some fluctuated without a clear trend (e.g. brick house with tiled/sheeted roof), others showed a downward trend (e.g. having a radio or a car), while others increased (e.g. private flush or Blair toilet) (Table 1). Average household size varied from 3.9 between 2003 and 2005 to 4.3 between 2009 and 2011. The proportion of household members who were male decreased over the survey period from 53.5 to 46.6%.

**Table 1:** Asset characteristics at the household level by study rounds and by quintiles (Round 5 only)

	Round 1	Round 3	Round 4	Round 5	Wealth quintile (Round 5)				
		
Indicator	(1998 to 2000)	(2003 to 2005)	(2006 to 2008)	(2009 to 2011)	First	Second	Third	Fourth	Fifth
Number of HH members									
Mean/median	4.1/4	3.9/4	4.1/4	4.3/4	4.6/4	4.5/4	4.4/4	3.9/4	4.3/4
Minimum/maximum	1/24	1/25	1/21	1/19	1/15	1/16	1/19	1/13	1/15
Proportion of men in HH (mean)	53.5	46.9	47.0	46.6	45.9	44.5	44.1	47.3	49.6
Non-sellable assets (%)									
Water piped into residence	6.9	5.3	6.2	5.8	0.4	1.4	0.6	13.0	12.3
Private flush or Blair toilet	50.7	60.1	63.0	60.9	25.6	38.7	93.6	58.6	81.6
Electricity	10.4	9.8	10.8	9.4	0.0	0.4	0.2	1.7	34.8
Brick house – tiled/sheeting roof	67.4	63.3	73.7	72.1	14.2	56.5	96.9	91.0	96.2
Finished floor (wood/cement/carpet)	81.0	80.4	81.7	76.2	6.2	76.9	96.8	96.2	98.6
Sellable assets (%)									
Radio	53.2	42.6	33.3	25.2	1.6	7.7	3.8	16.4	77.4
Television	11.2	13.8	13.0	11.4	0.1	0.4	0.5	1.6	42.5
Bicycle	15.2	18.6	15.7	11.9	1.4	3.4	2.5	8.0	35.5
Motorbike	1.2	0.5	0.6	0.4	0.0	0.0	0.0	0.1	1.5
Car	3.7	2.8	2.2	1.1	0.0	0.0	0.0	0.3	4.2
Combined asset score (mean)	0.379	0.372	0.371	0.340	0.134	0.264	0.332	0.365	0.541

HH, household.

All specific assets showed a clear difference by wealth level (Table 1) with greater proportions of wealthier households possessing each form of asset. For instance, households with a motorbike or car were only observed for the fourth and fifth quintile, while private flush or Blair toilets were recorded in one out of four households of the first quintile but in more than 80% of the fifth quintile.

**Figure 2:**
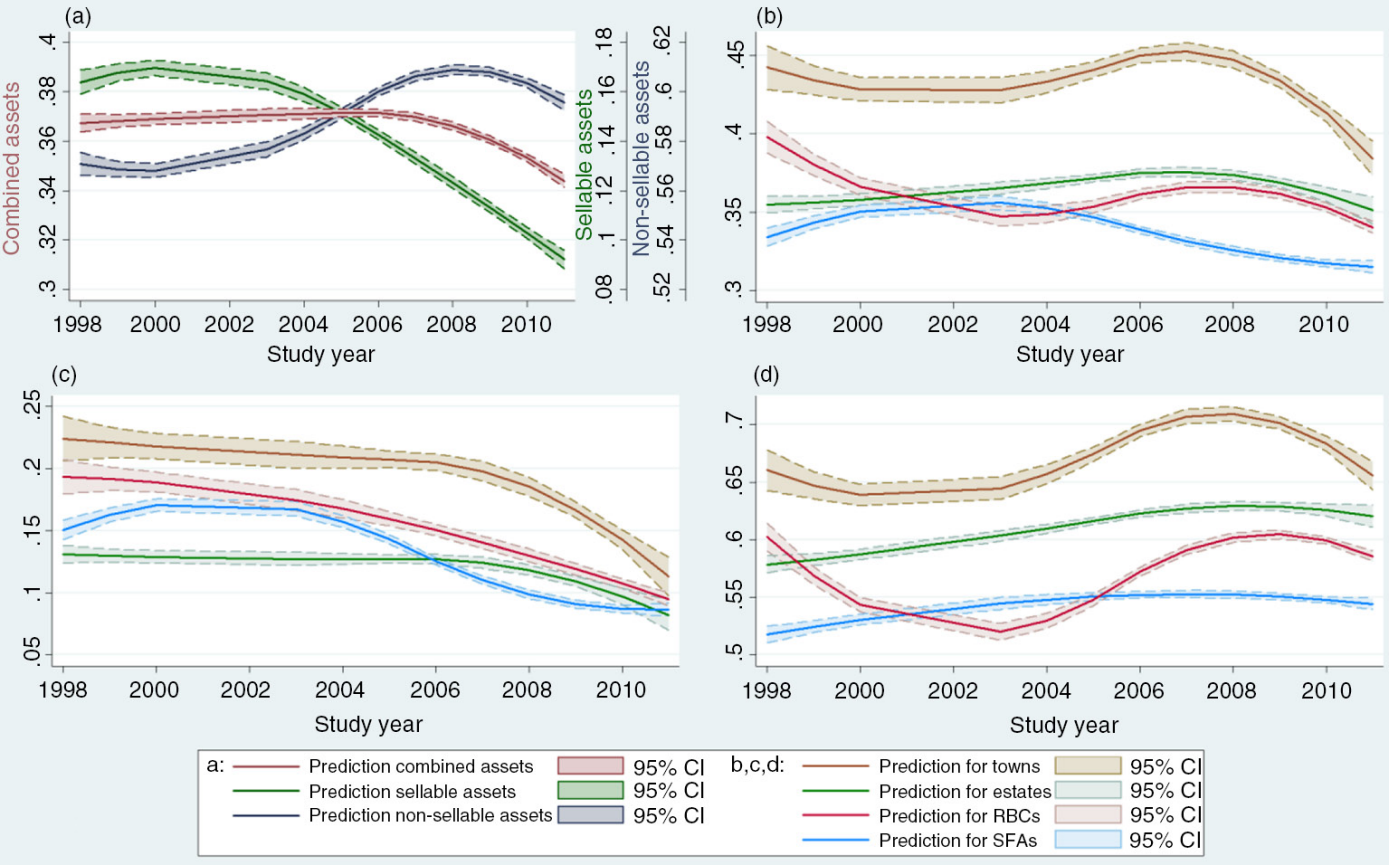
Trends in summed asset ownership score. Trends by (a) asset type, (b) site type for combined assets, (c) site type for sellable assets and (d) site type for non-sellable assets based on normal panel data regressions with quadratic B-spline functions of survey year.

At all rounds, the combined asset ownership score roughly followed a normal distribution that was slightly skewed to the right by visual assessment of histograms and Q-Q plots. The score was 0.369 initially and remained similar until 2007 but dropped significantly to below 0.350 in 2009 to 2011 (Figure 2a). Possession of sellable assets decreased steadily from 2003, in line with the national decline in GDP, but possession of non-sellable assets continued to increase until 2008 before falling significantly (Figure 2a). An additional analysis based only on balanced data showed very similar outcomes (results not presented).

Levels and trends in asset scores varied substantially between locations (Figure 2b). Asset scores were highest in small towns where reductions in sellable (Figure 2c) and non-sellable (Figure 2d) assets occurred only as the economy entered the hyper-inflation period (i.e. 2008). Households in SFAs had relatively few assets and appear to have been affected earliest by the economic decline, particularly with regard to possession of sellable assets. Households in RBCs showed trends intermediate to small towns and SFAs, reflecting the inclusion of a mixture of more urban and more rural households in these sites. Households on agricultural estates, generally, were less affected by the macro-economic changes, presumably because many of these households were owned and maintained by large private or parastatal companies and were occupied by estate workers who continued to be in paid employment. This situation was modified somewhat during the period of hyper-inflation when, in some instances, cash incomes were replaced with groceries and other forms of payment in kind.

By 2011, the gap in sellable assets owned between households in the different socio-economic locations was much smaller than at the start of the survey in 1998, and the formerly significant differences between estates, RBCs and SFAs had disappeared (Figure 2c). Large differences remained, however, in non-sellable assets (Figure 2b).

Specific assets contributed to the trends in asset ownership scores between socio-economic locations and survey rounds. The assets that varied the most were radios and type of toilet facility. For all locations, there was a significant decrease in the number of households owning a radio (contributing to the negative trend in sellable assets) from an average of 53.2% in 1998 to 2000 to 25.2% in 2009 to 2011. At the same time there was an increase in the number of households with private flush or Blair toilets (contributing to the positive trend in non-sellable assets) from 50.7 to 60.9% on average. Reductions in radio ownership were most pronounced in the more rural areas (RBCs and SFAs). Trends in other assets were less consistent and distinct.

**Figure 3:**
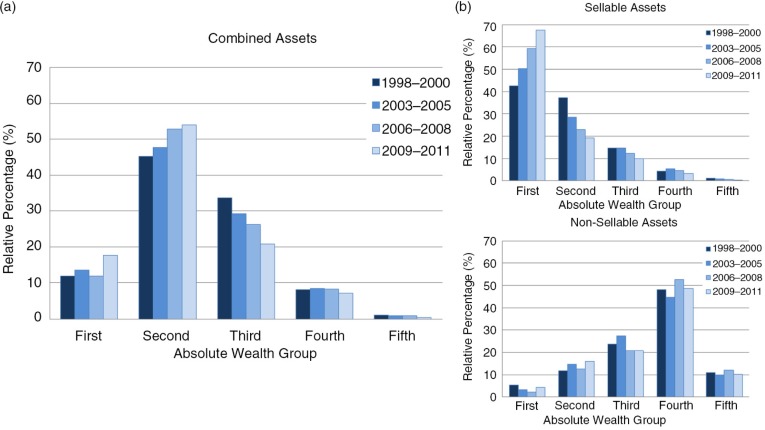
Absolute wealth groups over study rounds by asset type. (a) Combined assets; (b) sellable and non-sellable assets.

The overall reduction in household wealth, based on the combined asset scores, was not due to a general decrease in assets across all absolute wealth groups but arose mainly from a shift of households initially ranked in the third poorest wealth group into the second poorest group (Figure 3a). The proportion of households assigned to other absolute wealth groups remained fairly stable over time, except in 2009 to 2011, when more households were found to be in the poorest absolute wealth group than in any other round. While the distribution of sellable assets became more positively skewed over time (i.e. a reduction in sellable assets), the distribution for non-sellable assets remained more or less constant (Figure 3b). Due to small numbers of households in the fifth absolute wealth group, this group was combined with the fourth group to form a single group of highest absolute wealth in all subsequent analyses.

### Individual-level analysis

#### Socio-economic location

HIV prevalence in individuals aged 15 to 44 years living in households with complete wealth index data decreased steadily over time from 18.2 to 10.7% in men (Table 2) and from 25.5 to 16.8% in women (Table 3). This decline in HIV prevalence was observed in all socio-economic locations.

**Table 2:** Patterns in HIV prevalence by socio-economic characteristic and study round in men

	Round 1 (1998 to 2000)			Round 3 (2003 to 2005)			Round 4 (2006 to 2008)			Round 5 (2009 to 2011)		
				
Characteristics	*N*	HIV+ (%)	OR adjusted^a^	*N*	HIV+ (%)	OR adjusted^a^	*N*	HIV+ (%)	OR adjusted^a^	*N*	HIV+ (%)	OR adjusted^a^
Absolute wealth group												
First	369	23.0 (18.7–27.3)	1	654	16.4 (13.5–19.2)	1	468	12.6 (9.6–15.6)	1	856	10.5 (8.5–12.6)	1
Second	1620	17.5 (15.6–19.3)	0.91 (0.67–1.24)	2436	14.0 (12.6–15.3)	0.90 (0.66–1.23)	2239	11.5 (10.2–12.8)	1.06 (0.74–1.51)	2348	10.1 (8.9–11.4)	1.28 (0.91–1.81)
Third	1452	17.9 (15.9–19.9)	1.04 (0.76–1.43)	1835	13.0 (11.5–14.6)	0.83 (0.60–1.15)	1319	12.0 (10.2–13.7)	1.01 (0.69–1.48)	1127	11.4 (9.5–13.2)	1.29 (0.88–1.91)
Highest	426	18.1 (14.4–21.7)	0.99 (0.67–1.49)	643	14.6 (11.9–17.4)	0.94 (0.63–1.42)	467	11.8 (8.9–14.7)	0.93 (0.57–1.51)	404	12.9 (9.6–16.1)	1.35 (0.81–2.27)
Study site												
Small towns	721	26.4 (23.1–29.6)	1	956	21.7 (19.0–24.3)	1	763	16.4 (13.8–19.0)	1	823	13.9 (11.5–16.2)	1
Estates	1528	18.1 (16.1–20.0)	0.63* (0.49–0.80)	1824	15.6 (14.0–17.3)	0.55* (0.42–0.73)	1246	14.0 (12.1–16.0)	0.58* (0.42–0.80)	1339	10.6 (9.0–12.3)	0.57* (0.39–0.83)
RBC	513	15.8 (12.6–18.9)	0.68* (0.48–0.96)	1046	10.2 (8.4–12.1)	0.51* (0.36–0.72)	905	9.0 (7.1–10.8)	0.54* (0.37–0.78)	966	9.5 (7.7–11.4)	0.75 (0.50–1.11)
SFA	1105	14.3 (12.2–16.4)	0.56* (0.43–0.75)	1742	10.4 (9.0–11.8)	0.44* (0.32–0.60)	1579	9.4 (7.9–10.8)	0.50* (0.36–0.70)	1607	10.0 (8.5–11.4)	0.83 (0.58–1.19)
Occupation type												
Unemployed	825	15.5 (13.0–18.0)	1	1499	13.8 (12.1–15.6)	1	1694	11.9 (10.4–13.5)	1	1638	11.2 (9.7–12.8)	1
Unskilled (other)	1692	17.4 (15.6–19.2)	0.88 (0.66–1.18)	2371	10.3 (9.1–11.5)	0.80 (0.61–1.05)	1700	9.4 (8.0–10.7)	1.06 (0.80–1.39)	1677	7.2 (5.9–8.4)	1.35 (0.96–1.91)
Unskilled (informal)	756	18.8 (16.0–21.6)	0.80 (0.59–1.08)	982	18.2 (15.8–20.6)	0.97 (0.74–1.28)	579	16.1 (13.1–19.1)	1.15 (0.85–1.56)	784	13.4 (11.0–15.8)	0.99 (0.71–1.37)
Professional/skilled	594	23.6 (20.2–27.0)	0.63* (0.45–0.86)	716	20.9 (18.0–23.9)	0.69* (0.50–0.94)	520	14.4 (11.4–17.4)	0.63* (0.45–0.90)	636	15.6 (12.7–18.4)	0.84 (0.58–1.22)
Total	3867	18.2 (17.0–19.4)		5568	14.0 (13.1–14.9)		4493	11.8 (10.8–12.7)		4735	10.7 (9.8–11.6)	

a Adjusted for household-level clustering and controlled for potential confounding in absolute wealth group, study site, occupation type, age, education level, and marital status.

* Significant difference from reference category (*p*<0.05).

RBC, roadside business centres; SFA, subsistence farming areas, OR, odds ratio.

**Table 3:** Patterns in HIV prevalence by socio-economic characteristic and study round in women

	Round 1 (1998 to 2000)			Round 3 (2003 to 2005)			Round 4 (2006 to 2008)			Round 5 (2009 to 2011)		
				
Characteristics	*N*	HIV+ (%)	OR adjusted^a^	*N*	HIV+ (%)	OR adjusted^a^	*N*	HIV+ (%)	OR adjusted^a^	*N*	HIV+ (%)	OR adjusted^a^
Absolute wealth group												
First	614	30.5 (26.8–34.1)	1	1135	24.6 (22.1–27.1)	1	759	20.6 (17.7–23.4)	1	1284	15.1 (13.1–17.1)	1
Second	2278	26.1 (24.3–27.9)	0.95 (0.75–1.21)	3661	20.2 (18.9–21.5)	0.83 (0.68–1.00)	3066	18.7 (17.3–20.0)	0.87 (0.67–1.13)	3492	17.7 (16.4–19.0)	1.34* (1.07–1.68)
Third	1645	23.3 (21.3–25.4)	0.82 (0.63–1.06)	2345	19.7 (18.1–21.4)	0.84 (0.68–1.03)	1687	19.0 (17.1–20.8)	0.87 (0.65–1.16)	1467	17.2 (15.3–19.2)	1.33* (1.02–1.73)
Highest	495	23.8 (20.1–27.6)	0.70* (0.49–0.99)	795	20.1 (17.3–22.9)	0.76 (0.58–1.01)	622	18.8 (15.7–21.9)	0.74 (0.51–1.08)	584	14.4 (11.5–17.2)	0.90 (0.62–1.30)
Study site												
Small towns	721	43.5 (40.0–47.1)	1	956	29.4 (26.9–32.0)	1	763	29.5 (26.6–32.5)	1	823	22.2 (19.9–24.5)	1
Estates	1528	25.1 (22.8–27.4)	0.35* (0.27–0.46)	1824	21.1 (19.4–22.8)	0.56* (0.45–0.68)	1246	19.8 (17.8–21.7)	0.42* (0.32–0.55)	1339	17.0 (15.2–18.8)	0.54* (0.42–0.69)
RBC	513	22.8 (20.3–25.4)	0.31* (0.23–0.42)	1046	19.3 (17.4–21.2)	0.51* (0.40–0.64)	905	16.8 (14.7–18.9)	0.36* (0.26–0.48)	966	15.9 (14.0–17.9)	0.58* (0.44–0.75)
SFA	1105	14.3 (12.2–16.4)	0.27* (0.20–0.36)	1742	10.4 (9.0–11.8)	0.46* (0.37–0.56)	1579	9.4 (7.9–10.8)	0.35* (0.27–0.46)	1607	10.0 (8.5–11.4)	0.58* (0.44–0.75)
Occupation type												
Unemployed	2250	26.7 (24.9–28.5)	1	4353	21.5 (20.3–22.7)	1	4101	19.7 (18.5–20.9)	1	4526	17.9 (16.8–19.0)	1
Unskilled (other)	1031	17.4 (15.0–19.7)	0.74* (0.57–0.95)	1881	13.6 (12.0–15.1)	1.10 (0.90–1.35)	1295	11.6 (9.8–13.3)	0.92 (0.70–1.21)	1329	10.8 (9.1–12.4)	1.24 (0.94–1.65)
Unskilled (informal)	1549	28.4 (26.2–30.7)	0.96 (0.81–1.15)	1450	26.8 (24.5–29.1)	1.12 (0.95–1.32)	457	29.8 (25.6–34.0)	1.35* (1.01–1.80)	744	19.6 (16.8–22.5)	0.95 (0.74–1.22)
Professional/skilled	202	31.2 (24.8–37.6)	0.97 (0.66–1.43)	252	24.6 (19.3–29.9)	0.87 (0.61–1.24)	281	25.6 (20.5–30.7)	0.96 (0.66–1.39)	228	22.4 (16.9–27.8)	1.02 (0.67–1.54)
Total	5032	25.5 (24.3–26.7)		7936	20.7 (19.8–21.6)		6134	19.0 (18.0–20.0)		6827	16.8 (15.9–17.7)	

a Adjusted for household-level clustering and controlled for potential confounding in absolute wealth group, study site, occupation type, age, education level, and marital status.

* Significant difference from reference category (*p*<0.05).

RBC, roadside business centres; SFA, subsistence farming areas, OR, odds ratio.

The proportion of the population with secondary or higher education increased from 62% in 1998 to 2000 to 81% in 2009 to 2011, but the ranking between socio-economic locations remained constant, with the highest to lowest proportions being found in RBCs, SFAs, small towns and estates. In 2009 to 2011, individuals with secondary or higher education lived in wealthier households, with an average asset score of 0.358 compared to 0.285; 92% of men and women living in the wealthiest category of households (based on absolute wealth scores) had at least secondary school education compared to 68% of those living in the poorest households. HIV infection risk in men and women was significantly lower among those with at least secondary education in all time periods.

In all survey rounds and for both sexes, living in a town was associated with significantly greater risk of HIV infection after adjusting for education level and other demographic factors, except for men in 2009 to 2011, when no significant difference was observed compared to men living in the most rural areas (Table 2).

#### Absolute wealth

In men, HIV prevalence did not differ by household wealth (before and after adjusting for education and other confounding factors). Statistically significant reductions in HIV prevalence were recorded between 1998 to 2000 and 2009 to 2011 in all absolute wealth groups except the highest wealth group, for which a non-significant increase was recorded between 2006 to 2008 and 2009 to 2011 (Table 2). The lack of association between household wealth and HIV prevalence was also observed for sellable and non-sellable assets when examined separately (results not shown).

In women, at baseline (1998 to 2000), there was a tendency towards lower HIV prevalence with increasing household assets and, after adjusting for confounding factors, those living in the wealthiest households had significantly reduced HIV prevalence (Table 3). Between 1998 to 2000 and 2009 to 2011, the variation in prevalence between household wealth groups narrowed and significant declines were observed in all absolute wealth groups, with the largest and second largest reductions being found in the poorest and wealthiest groups, respectively. Multivariate logistic regression models showed that, after adjusting for confounding factors, women in the highest wealth group were at the lowest risk of infection in all rounds of the survey (Table 3), but the difference compared to women in the poorest households was only statistically significant at baseline. In 2009 to 2011, women of moderate wealth (in the second and third poorest groups of households) had significantly higher HIV prevalence compared to those from the poorest households.

Based on absolute levels of sellable assets (results not shown), women in the third poorest group of households were significantly less likely to be HIV positive than those in the poorest households up to 2005. The same was true for women from the wealthiest households in 1998 to 2000 and 2006 to 2008. For non-sellable assets, women from the wealthiest households showed statistically non-significant negative associations with HIV prevalence.

#### Occupation

HIV prevalence was highest in men in professional/skilled employment in most rounds of the survey but this difference was reversed after controlling for confounding factors, with the protective effect compared to unemployed men being statistically significant until 2006 to 2008 (Table 2). Unemployed men had the lowest HIV prevalence in 1998 to 2000 and the second lowest HIV prevalence from 2003 onwards (with other unskilled labour having the lowest prevalence). For women, no consistent pattern of association between HIV prevalence and occupation was observed in the data (Table 3).

Statistically significant declines in HIV prevalence between 1998 to 2000 and 2009 to 2011 were recorded for men in all occupations and for women in all occupations except professional/skilled employment.

Omitting occupation from the multivariate models had only marginal effects on the results for the remaining variables and did not affect significance in the absolute wealth groups (results not shown).

## Discussion

In this paper, we described patterns of association between socio-economic status (place of residence, household wealth and occupation) and HIV prevalence in areas of eastern Zimbabwe in the context of a major national economic decline. In general, individuals living in the most developed areas (small towns) had the highest levels of HIV prevalence; those living in households with greater and smaller numbers of assets had similar HIV prevalence; and those with occupations associated with greater and smaller incomes also had similar HIV prevalence.

For women, there was some evidence in the data during the earlier part of the study period (1998 to 2008) to support an association between greater household poverty and higher HIV prevalence. However, for men, there was little evidence for a positive association between wealth or income and HIV prevalence. In fact, being in professional or other skilled employment was associated with lower HIV prevalence at baseline (1998 to 2000), suggesting that any elevation in infection rates early in the epidemic had already been reversed.

Parkhurst [1] presented a comparison of HIV prevalence and relative wealth in 12 sub-Saharan African countries based on national income. He found that, in countries with GDP *per capita* below US$2000, HIV prevalence generally increased in line with wealth, but that, in richer countries, there was no clear pattern. In the study, Zimbabwe had a GDP just above US$2000. In contrast to our findings, using data from the national Demographic and Health Survey (DHS) 2005/06, Parkhurst found that female HIV prevalence increased with wealth up to the fourth quintile; however, he also found that prevalence was lowest in the wealthiest group. No associations were found for men. His results may have been confounded by effects of socio-economic location and other related factors such as circular labour migration [38].

Strengths of the current study include the large general population sample collected in areas where HIV levels are similar to those for the country as a whole [24] and the availability of longitudinal data that span the period of economic crisis in Zimbabwe and allow assessment of changes in wealth and HIV prevalence over time. For consistency with earlier studies and to establish how the burden of infection compares between socio-economic groupings, we examined associations between wealth and HIV prevalence. However, a limitation of the study is that we were not able to determine whether any significant associations observed were caused (for example) by wealth or whether HIV serostatus was, in fact, altering wealth. Furthermore, prevalence is a cumulative measure that changes slowly over time and is insensitive to short-term effects. Altering trends following the stabilization in Zimbabwe's economy in 2009 might not be observable yet, given that our most recent HIV prevalence data were collected between 2009 and 2011.

HIV incidence and mortality, which mediate changes in HIV prevalence, are potentially more directly linked to wealth and more sensitive to economic trends. In an analysis of data from the first two rounds of the Manicaland study, Gregson *et al*. [32] showed that the overall reduction in prevalence was not due purely to high AIDS mortality but also reflected behavioural changes, such as reductions in casual sex and delayed sexual debut, likely to have reduced HIV incidence. In the same period, Lopman *et al*. [31] found some evidence for negative associations between household wealth and both HIV incidence and mortality. However, these associations may have altered over time, for example due to subsequent differences in sexual behaviour change or uptake of ART services. More generally, surprisingly few studies have investigated associations between wealth and HIV incidence and mortality. Studies of this kind would be useful *inter alia* for assessing socio-economic differentials in access to prevention and treatment services.

We used panel data to take into account repeated observations of the same households and individuals in different survey rounds using regressions with fixed random effects. Our models assumed that missing observations occurred randomly but households and individuals might have dropped out or refused to be interviewed in follow-up surveys because of factors related to the outcomes. However, the probability of follow-up was similar across wealth groups, making it unlikely that our results are biased with respect to wealth. With respect to HIV serostatus, follow-up rates in HIV positives were higher than in HIV negatives across all rounds so we may overestimate HIV infection risk.

We used an index of absolute levels of household assets, which permits examination of differences and changes in the distribution of wealth and of changes in the association between household wealth and HIV prevalence over time. However, wealth calculated as a simple summed asset score is a rather crude measure, with each item contributing equally to the final estimate. Multiple correspondence analyses could be used instead to create a more sophisticated measure based on the first dimension of the correlation matrix [37]. Exploratory analyses showed (results not presented) that the correlation between the two methods was high (R^2^≥0.93) for each study site type. Therefore, we assumed that both methods are equally good for creating a wealth measure and chose the simpler method.

For consistency with a previous publication covering the initial period of the study [31], the asset index implemented in this paper is based on 10 different assets measured in the household census questionnaire. A validated standardized asset index score (the Progress out of Poverty Index) has been proposed to facilitate greater comparability between settings [39]. However, this index was not launched until 2005 (i.e. after the beginning of the current study) and, to our knowledge, the necessary income survey and validation of potential asset index questions have not yet been implemented in Zimbabwe.

Asset-based approaches may be inferior at capturing individual wealth. The household head might be in sole charge of financial decisions so that other household members depend on his/her generosity. Household wealth is taken as being the same for each member but individual wealth could be very different. In the absence of direct data on incomes, occupation might be a better proxy than household wealth for individual wealth. We found that men in professional employment were at lower HIV risk than unemployed men until 2006 to 2008 after accounting for confounding and wealth group. However, we did not observe any consistent associations in women, possibly because these differences were obscured by high rates of transition between occupations over survey rounds and differences in the husband's income for married women. A multivariate analysis of data from the Tanzania HIV/AIDS Indicator Survey 2003–04 [13,40] also found lower HIV infection rates in men and similar rates in women between professional employment and unemployment. These results emphasize the importance of capturing individual wealth when analyzing the HIV-wealth relationship, especially in men.

Wealth estimated at household-level is likely to vary with the numbers of income generators and dependents within a household, and the interpretation of a given level of household wealth will differ greatly, for example, depending on whether it is a single-person household or a household with multiple income generators and several dependents. Adjusting the analysis for these factors would remove any related effects and provide a more realistic insight into the actual wealth of each household. However, information on income was not collected in the Manicaland study and employment data were only collected at the individual level for the subset of household members that participated in the individual cohort. Hence, the number of income generators and dependents within a household could not be obtained. Our individual-level analyses were adjusted for employment but employment is not the only way to generate money in this population. Income may also be obtained from remittances received from relatives working and living elsewhere (including the diaspora) or from selling goods or services in the informal sector. The latter may include sex work; however while data on sex work were collected in the study, levels reported in household surveys are typically underestimates so no attempt was made to adjust for these in the current analysis.

As drug supplies improve and treatment guidelines change, more people will have access to ART and survival will increase. Wealthier people might benefit more from these changes, which could lead to changes in associations between wealth and HIV prevalence. In 2009 to 2011, uptake of ART among self-reported HIV positives was only 16.3 and 26.9% in HIV-positive men and women, respectively, according to the results of our individual-level questionnaire. Women showed a positive statistical correlation between ART uptake and increasing household wealth in multivariate models (*p*=0.031). However, no significant differences in uptake were found between household wealth groups for men and occupation for either sex.

## Conclusions

We found that, for both sexes, HIV prevalence fell substantially between 1998 to 2000 and 2009 to 2011 in almost all socio-economic locations and sub-groups. Contrary to earlier concerns expressed by Lopman *et al*. [31] based on data from the Manicaland study from 1998 to 2000 and 2001 to 2003, HIV has not become increasingly a disease of the poor in eastern Zimbabwe. While the national economic decline was reflected in more extensive poverty in the study areas, the lack of strong associations between lower wealth and income and reduced HIV prevalence suggests that the economic decline may not have been a major factor driving the fall in HIV prevalence.
